# Effects of Cannabidiol on Bone Health: A Comprehensive Scoping Review

**DOI:** 10.3390/biomedicines14010208

**Published:** 2026-01-18

**Authors:** Shabbir Adnan Shakir, Kok-Yong Chin

**Affiliations:** Department of Pharmacology, Faculty of Medicine, Universiti Kebangsaan Malaysia, Bandar Tun Razak, Kuala Lumpur 56000, Malaysia; shabbir.md.shakir@gmail.com

**Keywords:** bone remodelling, CB2 receptor, osteoblast, osteoclast, periodontitis

## Abstract

**Background/objectives:** Cannabidiol (CBD) is a non-psychoactive constituent of *Cannabis sativa*, which has potential skeletal benefits through modulation of bone cell function and inflammatory signalling. However, evidence of its effects and mechanisms in bone health remains fragmented. This scoping review summarised the current findings on the impact of CBD on bone outcomes and its mechanisms of action. **Methods:** A systematic search of PubMed, Scopus, and Web of Science was conducted in October 2025 for original studies published in English, with the primary objective of examining the effects of CBD on bone health, regardless of study design. After applying inclusion and exclusion criteria, 24 primary studies were included. Data on model design, CBD formulation, treatment parameters, bone-related outcomes, and proposed mechanisms were extracted and analysed descriptively. **Results:** Among the studies included, eleven demonstrated beneficial effects of CBD on bone formation, mineralisation, callus quality, or strength; eleven showed mixed outcomes; and two demonstrated no apparent benefit. Previous studies have shown that CBD suppresses bone resorption by reducing osteoclast differentiation and activity while promoting osteoblast proliferation and matrix deposition. Mechanistically, CBD’s effects involve activation of cannabinoid receptor 2, modulation of the receptor activator of nuclear factor-κB ligand/osteoprotegerin pathway, and regulation of osteoblastogenic and osteoclastogenic signalling through bone morphogenetic protein, Wnt, mitogen-activated protein kinase, nuclear factor-κB, and peroxisome proliferator-activated receptor signalling. The anti-inflammatory and antioxidant actions of CBD further contribute to a favourable bone microenvironment. **Conclusions:** Preclinical evidence suggests that CBD has a bone-protective role through multifaceted pathways that enhance osteoblast function and suppress osteoclast activity. Nevertheless, robust human trials are necessary to confirm its efficacy, determine its optimal dosing, and clarify its long-term safety.

## 1. Introduction

Cannabidiol (CBD) is the principal non-psychoactive constituent of *Cannabis sativa* L. and has attracted considerable scientific interest because of its broad pharmacological profile and absence of the psychotropic effects characteristic of Δ9-tetrahydrocannabinol (THC) [1,2]. CBD exhibits antioxidant, anti-inflammatory, analgesic, and neuroprotective properties that have been explored across various neurodegenerative, metabolic, pain, and inflammatory disorders [3,4]. Emerging evidence suggests that these actions extend to the skeletal system, where the endocannabinoid system (ECS) plays a role in regulating bone formation, remodelling, and repair [5,6]. Because bone is a dynamic tissue that is continuously remodelled through osteoblastic formation and osteoclastic resorption, compounds that modulate this balance may have therapeutic value in conditions such as osteoporosis, periodontitis, and non-union fractures [7,8,9].

The ECS comprises the classical G-protein-coupled receptors cannabinoid receptor CB1 and CB2, endogenous ligands such as anandamide and 2-arachidonoylglycerol, and the enzymes responsible for their synthesis and degradation [2,10]. Both CB1 and CB2 are expressed in bone, with CB1 predominating in peripheral nerves and osteoclasts, and CB2 being expressed in osteoblasts, osteocytes, and stromal cells [11,12]. Activation of CB2 supports bone formation and limits osteoclastogenesis, whereas CB1 overstimulation favours bone resorption [13]. CBD acts as a weak inverse agonist at both CB1 and CB2, but also interacts with non-cannabinoid targets, including transient receptor potential vanilloid 1 (TRPV1), serotonin 5-hydroxytryptamine receptor 1A (5HT1A), peroxisome proliferator-activated receptor-γ (PPARγ), and G protein-coupled receptor 55 (GPR55) [14]. These multi-receptor interactions suggest that CBD can modulate oxidative stress, inflammatory mediators and differentiation pathways [15], resulting in a net improvement in bone health.

Preclinical studies report that CBD enhances bone regeneration and prevents osteolysis in several experimental models. In murine fracture models, systemic or local administration of CBD increased callus size, bone mineral density (BMD), and the mechanical strength of healing bone [12,16]. CBD has been shown to stimulate lysyl hydroxylase activity in osteoblasts, thereby improving collagen cross-linking and fracture stability [11]. In ovariectomised and spinal cord injury models that mimic post-menopausal and disuse osteoporosis, respectively, CBD prevented trabecular bone loss, decreased osteoclast numbers and normalised the receptor activator of nuclear kappa-B (RANKL)/osteoprotegerin (OPG) ratio [17,18,19]. In vitro studies using mesenchymal stem cells (MSCs) and osteoblast-like cell lines consistently report that CBD promotes osteogenic differentiation, increasing alkaline phosphatase (ALP) activity, collagen type I expression, and mineralised nodule formation [20,21].

Multiple signalling mechanisms underlie CBD’s skeletal effects, with TRPV1 activation and downstream p38 mitogen-activated protein kinase (MAPK) and extracellular signal-regulated kinase (ERK) 1/2 phosphorylation linked to runt-related factor 2 (RUNX2) upregulation and osteoblast maturation [22]. In osteolytic and inflammatory contexts, CBD suppresses nuclear factor-κB (NF-κB) signalling and reduces pro-inflammatory cytokines, including tumour necrosis factor-α (TNF-α), interleukin-1β (IL-1β), and interleukin-6 (IL-6) [23,24]. CBD also downregulates Toll-like receptor (TLR)-4 signalling and diminishes reactive oxygen species (ROS) [25,26], potentially mitigating inflammation and oxidative stress-induced bone resorption. CBD also suppresses activator protein-1 (AP-1) and nuclear factor of activated T-cells (NFAT) [27], both critical signalling molecules in osteoclastogenesis.

While the therapeutic potential of cannabinoids in musculoskeletal health has been explored in several previous reviews [28,29], they primarily focused on the broader class of cannabinoids or medical cannabis, leaving the specific skeletal effects of CBD under-scrutinised. Given the rapid development of preclinical studies, from molecular signalling in various cell lines to diverse animal models of bone pathology, the current evidence base remains fragmented. This lack of synthesis prevents the translation of laboratory findings into standardised clinical protocols. A scoping review approach was selected for this study, as this approach is specifically designed to map the extent, range, and nature of research activity in a given field. It allows for the inclusion of heterogeneous study designs, including in vitro, in vivo, and preliminary human data, which is essential for clarifying the myriad mechanisms and varied outcomes of CBD with regard to bone health.

## 2. Methods

This scoping review was designed according to the framework of Arksey and O’Malley [30] and in compliance with the Preferred Reporting Items for Systematic Reviews and Meta-Analyses extension for scoping reviews (Table S1) [31]. The following steps were adopted: (1) identifying the research question; (2) identifying the relevant studies; (3) study selection; (4) charting the data; (5) collating, summarising, and reporting the results. The protocol of this scoping review has been registered in the Open Science Framework (Url: https://osf.io/ep5ws/) (assessed on 21 December 2025).

### 2.1. Identifying the Research Question

The current scoping review addressed the question, “What are the effects of CBD on bone health and its mechanisms of action?”, using the Population, Intervention, Comparator and Outcomes (PICO) framework. The “Population” refers to in vitro bone cell models (osteoblasts, osteoclasts, osteocytes, or other related cells), animal models of skeletal conditions (such as osteoporosis, osteolysis, and fracture healing), or patients at risk of bone loss. The “Intervention” refers to CBD, while the “Comparator” refers to the untreated control. The “Outcomes” refer to any bone health measures, such as densitometry, histology, biomechanics, bone remodelling markers, and signalling markers.

### 2.2. Identifying Relevant Studies

A literature search was performed on three electronic databases, i.e., PubMed, Scopus, and Web of Science, in October 2025, using the following search string: (Cannabidiol) AND (Bone OR BMD OR Osteop* OR Fracture* OR Osteoblast* OR Osteoc*). The search string was applied to titles and abstracts to avoid nonspecific results. All items between the inception of databases and the date of the search were included. In this search, all primary studies involving in vitro, in vivo, or human models that investigated the effects of cannabidiol on bone health were considered.

Articles without primary results, such as reviews, perspectives, commentary, letters to the editor, books, and book chapters were not considered. Conference abstracts and proceedings were not included due to incomplete data and potential overlap with the full reports. Articles not written in English were excluded. Studies using mixed formulations, for example, a mixture of CBD and THC or other compounds, in which individual effects of CBD could not be delineated, were excluded.

### 2.3. Study Selection

**Figure 1:**
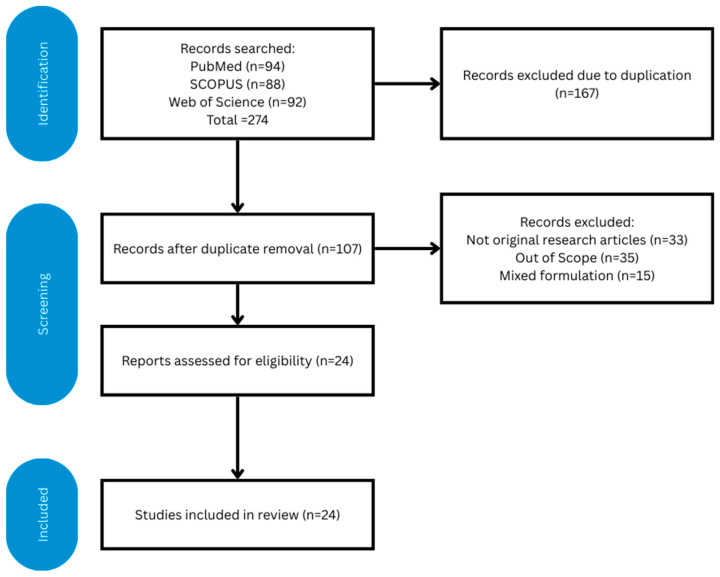
PRISMA flowchart on the article selection process [drawn using Canva (Surry Hills, NSW, Australia)].

The search results were downloaded from the three electronic databases. Duplicated items were removed using EndNote 2025 (Clarivate, Philadelphia, PA, USA), and the results were manually verified to ensure successful deduplication. The titles and abstracts were screened by two researchers (S.A.S. and K.-Y.C.) independently based on the inclusion and exclusion criteria. Next, the full texts of the eligible items were obtained and screened by the same researchers. The degree of agreement between the two researchers, represented by Cohen kappa values, was 0.97. Disagreements were resolved by discussion. The article selection process is summarised in Figure 1.

### 2.4. Charting the Data

S.A.S. and K.-Y.C. extracted relevant information from the selected studies, which included researchers, publication years, study design (subjects or disease models used, dosage of cannabidiol, treatment period), limitations, and major findings, using a standard Google Sheet (Google, Mountain View, CA, USA).

### 2.5. Collating, Summarising, and Reporting the Results

The results were summarised descriptively due to the heterogeneity of study designs, methods, outcome measures, and reporting formats across studies. Data synthesis based on statistical methods was not conducted because the studies varied significantly in terms of experimental design, CBD purity, delivery routes, and treatment duration. The effect of CBD on bone health, as well as the pathogenesis of these effects, current research gaps in the field, and limitations of this review, are discussed.

## 3. Results

### 3.1. Study Characteristics

A total of 24 primary studies met the inclusion criteria. They consisted of eight cell culture studies (33.3%), eight animal studies (33.3%), seven studies with both cell culture and animal models (29.2%) and one human clinical trial (4.2%). Publication years ranged from 2008 to 2024. Most studies investigated purified CBD, while a minority used CBD-loaded biomaterials (e.g., hydrogels, scaffolds) or CBD administered alongside standard osteogenic stimuli.

Among the cell culture studies (Table 1), the representative osteoblast models used included MC3T3-E1, U20S, and MG-63 cell lines, as well as primary cells from human adipose stem cells, murine calvarial tissue, and murine bone marrow mesenchymal stem cells. The sources of osteoclast-like cells were RAW 264.7 macrophages, murine bone marrow macrophages, and human peripheral blood macrophages. In dental studies, human periodontal ligament cells and dental pulp stem cells were used.

Among the animal studies (Table 2), the models used included fluoxetine, a vitamin D-deficient diet and ovariectomy-induced osteoporosis; spinal cord injury; ligature-induced periodontitis; fracture repair; and vertebral bone defects. One toxicity study used healthy dogs. The clinical study assessed the effects of long-term oral CBD consumption in healthy adults.

CBD dosing varied widely across studies, ranging from 0.1–10 µM in vitro to 5–20 mg/kg/day in vivo. In the in vitro studies with a CBD dose exceeding 10 µM murine periosteal progenitor cells, 12.5 µM was cytotoxic to human dental pulp stem cells, while 30 µM was cytotoxic to macrophages or osteoclast precursors. Treatment duration spanned from 24 h in cell culture studies to 8–12 weeks in animal models.

### 3.2. Effects of CBD on Bone Formation and Osteoblast Activity

Across the included in vitro studies, CBD consistently exhibited pro-osteogenic activity. Multiple studies demonstrated that CBD enhanced osteoblast or mesenchymal stem cell differentiation by increasing ALP activity, collagen type I deposition, and expression of RUNX2 and Osterix [16,21,22,32,37,39]. Enhanced mineralised nodule formation was frequently observed, indicating the promotion of late-stage osteoblast maturation [16,22,39]. CBD also increased cell viability and proliferation in several models, especially at lower doses. A clear dose-dependent effect of CBD in promoting osteogenic differentiation was noted in several studies [21,22]. Dose-dependent inhibitory effects of CBD on osteoblasts were only observed at higher concentrations [16,34].

Findings from biomaterial-based studies further supported CBD’s osteogenic effects. CBD-loaded hydrogels and scaffolds promoted osteoblast proliferation and supported robust bone-like tissue formation, outperforming unloaded controls [20,32].

Animal studies reinforced these pro-osteogenic findings. In models of fracture healing, ovariectomy-induced osteoporosis, bone defect repair, and spinal cord injury, CBD improved trabecular bone volume, trabecular thickness, and biomechanical strength [11,12,16,19]. In fracture models, CBD enhanced callus size as well as mineralisation, increased lysyl hydroxylase activity, promoted collagen cross-linking, and improved extracellular matrix stability [11]. Local CBD delivery methods (e.g., microspheres, hydrogels) produced particularly strong regenerative effects in critical-sized defects [20,32]. Two studies reported limited benefits, probably due to short treatment duration or insufficient CBD dosing [18,42].

### 3.3. Effects of CBD on Bone Resorption and Osteoclast Activity

Most studies investigating osteoclastogenesis demonstrated that CBD suppresses osteoclast differentiation and resorptive activity. In vitro, CBD decreased the formation of tartrate-resistant acid phosphatase (TRAP)-positive multinucleated osteoclasts and the formation of resorption pits [24,34,36]. These effects were associated with downregulation of RANKL-induced pathways and key transcription factors such as c-Fos and nuclear factor of activated T-cells, cytoplasmic 1 (NFATc1) [24,36]. CBD also attenuated pro-inflammatory cytokine signalling, reducing levels of TNF-α, IL-1β, and IL-6 through inhibition of NF-κB activation [23].

CBD’s antagonism of GPR55 was shown to reduce osteoclast activity and improve bone mass [40], while CB2-mediated signalling contributed to the suppression of osteoclast maturation [21,34]. Interestingly, CBD increased bone resorption of osteoclasts at a lower concentration (10 µM) but inhibited it at the same concentration when the osteoclasts were co-cultured with osteoblast-like cells [34].

In vivo studies validated these anti-resorptive effects. CBD reduced osteoclast numbers and restored the RANKL/OPG balance in models of oestrogen deficiency [16,42]. In experimental periodontitis, CBD greatly reduced bone resorption and inflammatory cytokine production [24]. Models of spinal cord injury also showed reduced activation of TLR4 and NF-κB. This highlights that CBD attenuates inflammation-driven bone degradation [19]. Neutral or minimal effects were observed only in short-duration or low-dose studies [18].

### 3.4. Human Clinical Evidence

Only one study meeting the inclusion criteria evaluated CBD’s effects on bone-related outcomes in humans. A long-term oral CBD administration study in healthy adults found the treatment to be well-tolerated, with no significant adverse events reported [44]. Although the study did not assess bone mineral density or structural parameters, a modest increase in serum bone-specific alkaline phosphatase (BALP) was noted. This suggests a potential osteogenic response. However, the lack of imaging data and the inclusion of only healthy participants without underlying bone pathology limit the translational value of this study on bone health. In conclusion, the clinical evidence is insufficient to draw meaningful conclusions regarding the effects of CBD on bone health in humans.

### 3.5. Mechanisms of Action Across Studies

The mechanistic findings across the included studies reveal a systematic pattern by which CBD influences bone remodelling. A central mechanism involves suppression of inflammatory pathways. Several studies demonstrated that CBD inhibited NF-κB activation and reduced pro-inflammatory cytokines, including TNF-α, IL-1β and IL-6 [23,24]. In inflammatory bone loss models, CBD also inhibited TLR4 signalling and decreased oxidative stress markers. This plays a role in the preservation of bone tissue [19].

CBD also activates pro-osteogenic signalling pathways. Studies have highlighted that CBD activates the ERK1/2 and p38 MAPK pathways, leading to increased expression of RUNX2, Osterix, and bone morphogenetic protein (BMP) 2/4 [21,22,37,39]. TRPV1 activation also contributes to osteogenic differentiation [22].

Interactions with the endocannabinoid system further explained CBD’s skeletal effects. Activation of CB2 receptors promoted osteoblast differentiation [21], while inhibition of CB1 receptors reduced bone resorption. Antagonism of GPR55, a receptor that promotes osteoclast formation, also contributed to CBD’s anti-resorptive actions [40].

These mechanisms suggest that CBD has a dual action, enhancing osteoblastogenesis while inhibiting osteoclast maturation. CBD supports these actions by decreasing inflammation and oxidative stress.

## 4. Discussion

This scoping review demonstrates that CBD primarily exhibits positive skeletal effects across preclinical models. It specifically induces increased osteoblast differentiation and reduced osteoclastogenesis. These findings align with earlier work demonstrating that the endocannabinoid system regulates bone homeostasis through CB1 and CB2 receptors, with CB2 activation generally promoting bone formation and inhibiting bone resorption [45,46,47,48].

**Figure 2:**
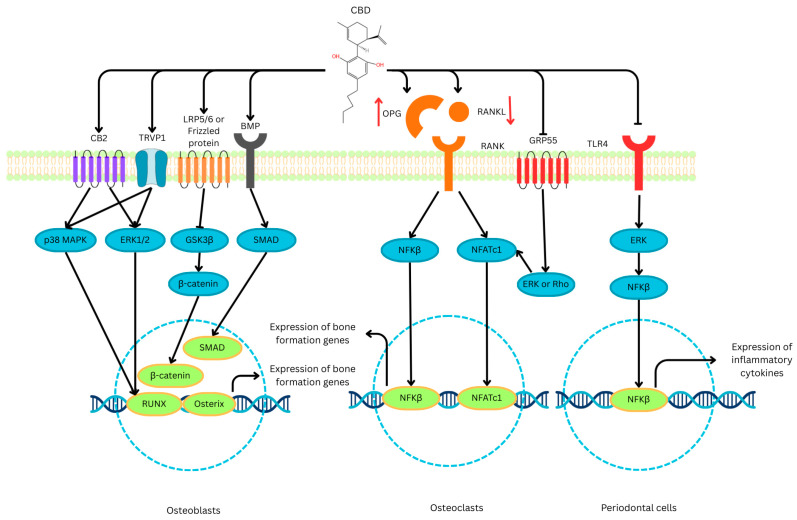
Molecular signalling pathways modulated by CBD in the skeletal system. CBD exerts a bone-protective effect through a triple-action mechanism: (**left**) stimulating osteoblast differentiation and bone formation via CB2, TRPV1, Wnt/β-catenin, and BMP signalling; (**centre**) suppressing osteoclastogenesis by modulating the RANKL/OPG ratio and inhibiting NF-κB/NFATc1 pathways; and (**right**) exerting anti-inflammatory effects in periodontal cells by downregulating TLR4-mediated cytokine expression. Black arrows indicate stimulation; T-bars indicate inhibition; red arrows indicate up/downregulation of protein levels [drawn using Canva (Surry Hills, NSW, Australia)]. Abbreviations: BMP, bone morphogenetic protein; CB2, Cannabinoid receptor type 2; CBD, cannabidiol; ERK1/2, extracellular signal-regulated kinases 1 and 2; GPR55, G protein-coupled receptor 55; GSK3β, glycogen synthase kinase 3 beta; LRP5/6, low-density lipoprotein receptor-related protein 5 and 6; NFATc1, nuclear factor of activated T-cells, cytoplasmic 1; NF-κB, nuclear factor kappa-B; NLRP3, NOD-like receptor family, pyrin domain-containing 3; OPG, osteoprotegerin; p38 MAPK, p38 mitogen-activated protein kinase; RANK, receptor activator of nuclear factor kappa-B; RANKL, receptor activator of nuclear factor kappa-B ligand; RUNX, runt-related transcription factor; SMAD, small mother against decapentaplegic; TLR4, Toll-like receptor 4; TRPV1, transient receptor potential vanilloid 1.

Across the studies reviewed, CBD increased alkaline phosphatase activity, RUNX2 and Osterix expression, and collagen type I deposition and improved trabecular microarchitecture and biomechanical properties [11,16,19]. In addition to promoting osteoblast activity, CBD consistently reduced osteoclast differentiation, as well as TRAP-positive multinucleated cells and c-Fos/NFATc1 signalling [24,36]. The effects of CBD on the bone health process, specifically focusing on the mechanism, are summarised in Figure 2.

The anti-inflammatory actions of CBD, through the inhibition of the NF-κB, TLR4, and NOD-like receptor family, pyrin domain containing 3 pathways, also contributed to reduced osteoclastogenesis [23,35]. Collectively, these results imply a dual, complementary role where CBD enhances bone formation while limiting bone resorption, resulting in a favourable remodelling balance.

Mechanistic data indicate that CBD modulates multiple signalling networks important for skeletal homeostasis. CBD activated pro-osteogenic pathways, including ERK1/2, p38 MAPK, BMP2/4, and Wnt/β-catenin, hence promoting RUNX2-driven osteoblast differentiation [21,22,39]. TRPV1 activation and downstream MAPK phosphorylation have also been noted in osteogenic commitment [22].

Anti-resorptive effects of CBD are mediated through suppression of the RANKL–RANK–NFATc1 axis and antagonism of GPR55, a receptor known to enhance osteoclast formation [40]. CBD has been shown to inhibit inflammatory mediators such as TNF-α, IL-1β, and IL-6 [23,24]. Moreover, CBD reduces oxidative stress [25]. Both effects significantly influence the behaviour of osteoblasts and osteoclasts.

Interaction with the broader endocannabinoid system provides an additional mechanistic layer. CB2 activation promotes bone formation, whereas excessive CB1 signalling favours resorption [45,46,47]. CBD functions as a weak inverse agonist at CB1 and CB2, and modulates the PPARγ, serotonin 5-HT_1A_, and TRP channels [49,50,51], which enables wide-ranging effects. Besides its weak orthosteric binding, studies have suggested that CBD acts as an allosteric ligand of CB2 [52], and as inverse antagonist and inducer of CB2 and 5-HT receptor heterodimerization [53]. However, the action through which CBD modulates bone cells remains unclear. These mechanistic pathways are consistent with recent reviews emphasising CBD’s multi-target pharmacology in bone, immune regulation, and tissue repair [5,54].

While the overall preclinical signal is positive, findings from a small number of studies suggest that the treatment duration, dosing, or delivery route of CBD may critically influence outcomes [17,18]. Moreover, the single eligible human study demonstrated acceptable tolerability of CBD, but no clear structural bone improvements [44]. Hence, clinical evidence remains insufficient. Nonetheless, the current evidence does indicate that CBD has mechanistic as well as functional properties with potential therapeutic relevance for conditions such as osteoporosis, periodontitis, and impaired fracture healing.

Evidence from human observational and clinical studies remains inconsistent. Some retrospective analyses suggest cannabis exposure involving THC impairs bone healing or reduces fusion success. However, these findings do not isolate the effects of CBD. The lack of controlled CBD-specific clinical trials limits conclusions about human its efficacy, a limitation noted widely in the musculoskeletal and cannabinoid literature [55].

From the studies included in this review, evidence of a CBD formulation that is clearly superior for clinical application appears to be insufficient. Preclinical data indicate that local delivery systems such as hydrogels, microspheres, or composite scaffolds enhance osteoconductivity, mineralisation, angiogenic responses, and antimicrobial properties of CBD compared with unloaded controls, supporting their potential advantages for site-specific bone regeneration [5,20,32,56,57]. These biomaterial-based strategies align with broader principles in regenerative medicine that emphasise targeted local delivery to maximise therapeutic efficacy while minimising systemic exposure [58]. In contrast, systemic administration of CBD via oral or intraperitoneal routes has been predominantly employed in models of osteoporosis, fractures, and inflammation-associated conditions. In these models, improvements in trabecular micro-architecture and bone strength have been observed [12,17,19]. However, the heterogeneity of experimental designs and the absence of head-to-head comparisons between delivery systems preclude clinical recommendation of any specific CBD formulation. This gap calls for comparative and translational studies in the future.

Sex is a key determinant of bone biology and may influence responses to pharmacological interventions [59]. The current literature demonstrated the beneficial effects of CBD on bone-related outcomes in both male and female preclinical models. In OVX models mimicking postmenopausal bone loss, improvements in trabecular microarchitecture and modulation of osteoclast-related pathways were reported [16,17,42]. Studies conducted in male animals, primarily using fracture or inflammation-associated models, have reported enhanced bone repair and reduced resorption [11,12,19,24]. However, no studies directly compared male and female responses under identical experimental conditions. Consequently, the available evidence does not permit firm conclusions regarding sex-specific effects of CBD on bone health. Future studies incorporating sex-stratified designs and head-to-head comparisons are necessary to determine whether biological sex influences the magnitude or mechanism of CBD’s skeletal effects.

Despite its promising therapeutic potential, the application of CBD in bone health faces significant scientific and clinical challenges. Isolation and purification remain problematic due to CBD’s lipophilic nature and chemical instability. The absence of standardised extraction protocols results in substantial variability in product quality and potency [60]. Difficulties in characterisation stem from the lack of validated analytical methods, and formulation development is hindered by CBD’s poor aqueous solubility and limited bioavailability [60,61]. Ethical challenges include regulatory uncertainties, informed-consent complexities given CBD’s association with cannabis, and the proliferation of unregulated commercial products [61].

Clinical administration of CBD requires careful consideration of dose-dependent adverse effects. While generally well-tolerated at therapeutic doses (up to 1500 mg/day for multiple doses, and 6000 mg for a single dose) [62], excessive CBD intake can cause hepatotoxicity, particularly when combined with other medications metabolised via CYP450 enzymes [63,64,65]. While the interactions between CBD and current anti-osteoporosis medications have not been evaluated, it is a critical aspect to be considered clinically. Essential clinical precautions for clinical use of CBD should include baseline and interval liver function testing, comprehensive medication reviews to identify interaction risks, initiation at low doses with gradual titration, use of pharmaceutical-grade CBD products with the highest purity, and patient education regarding potential adverse effects. However, until controlled human trials are conducted, the role of CBD in mitigating degenerative bone diseases remains speculative. For clinical translation, targeted phase I or II trials should evaluate CBD for fracture healing, implant osseointegration, or postmenopausal bone loss using standardised, objective endpoints (BMD, CT-based microarchitecture, and biochemical markers).

The strengths of the included studies in this review lie in the breadth of models examined, which include fracture healing, osteoporosis, spinal cord injury, and periodontal disease. The consistency of osteogenic and anti-resorptive responses across independent laboratories strengthens confidence in the underlying biological effects.

However, several limitations warrant caution. Many studies employed small sample sizes, lacked randomisation, or did not report methodological details. Dosing regimens varied widely, and some in vitro studies used supraphysiological concentrations that may not reflect achievable human exposures [32,34]. The heterogeneity of CBD formulations, which range from purified CBD to oil-based extracts or CBD-loaded biomaterials, complicates comparisons across studies. Importantly, very few studies provided pharmacokinetic data or examined long-term outcomes. Some animal models demonstrated minimal benefit due to inadequate dosing or short treatment windows [17,18]. These limitations are consistent with recognised challenges in cannabinoid-related bone research [55,66].

The strengths of this review include its comprehensive search strategy, its structured methodological approach following the Arksey and O’Malley and PRISMA-ScR guidelines, and the systematic charting of data across in vitro, animal, and human studies. The review also integrates mechanistic and functional outcomes, which increases our holistic understanding of CBD’s potential skeletal effects.

On the other hand, this scoping review also has its limitations. Only English-language publications were included, and conference abstracts were excluded, omitting possibly important evidence. Because scoping reviews aim to map the literature rather than synthesise effect sizes, no meta-analysis was conducted. Grey literature and unpublished negative studies were not searched; therefore, selection biases could not be excluded for this review.

Future work should focus on clinical translation. Firstly, pharmacokinetic studies should be conducted to define biologically active and clinically achievable CBD concentrations [67,68]. It would be highly beneficial for the standardisation of CBD formulations, specifically in terms of purity and delivery vehicles, to allow for reproducibility as well as regulatory approval [51,68]. Next, early-phase clinical trials should be conducted targeting high-need populations, such as patients with non-union fractures, postmenopausal women with osteopenia, or dental implant recipients [44,55].

As CBD continues to gain attention, interdisciplinary collaboration between orthopaedics, dentistry, biomaterials engineering, and cannabinoid pharmacology will be essential. Only through such integrative efforts can the true potential of CBD in musculoskeletal medicine be fully explored and translated into evidence-based therapies.

## 5. Conclusions

This scoping review demonstrates that CBD exerts multifaceted and predominantly positive effects on bone biology. CBD enhances osteoblast differentiation, supports matrix formation, and suppresses osteoclast-driven resorption. These effects are mediated through a network of anti-inflammatory, antioxidant, and receptor-dependent mechanisms. Although preclinical evidence consistently highlights CBD’s promise as a modulator of skeletal remodelling as well as a potential therapeutic candidate for conditions such as osteoporosis, fracture healing, and inflammatory bone loss, human data remain insufficient to guide clinical practice. Significant gaps persist regarding optimal dosing, delivery strategies, long-term safety and translational efficacy. As interest in CBD-based therapeutics continues to grow, rigorous clinical trials and standardised experimental approaches will be essential to determine whether CBD can evolve from a biologically compelling compound into a clinically actionable treatment for bone-related disorders.

## Figures and Tables

**Table 1:** Summary of cell culture studies on the effects of CBD on skeletal health.

Authors (Year)	Subject and Model Characteristics	Treatment Characteristics	Findings		
			Increased vs. Negative Control	Decreased vs. Negative Control	Unchanged vs. Negative Control
Baines et al. (2025) [32]	MC3T3-E1 cells seeded on 40% (*w*/*v*) whey protein isolate (WHI) hydrogels.	CBD in hydrogels at 10–50 µM. Negative controls included WPI hydrogels without CBD. Positive control: n/a.	Mechanical properties. Cell proliferation. ALP and collagen protein expression.	Hydrogel mass loss.	Hydrogel swelling mass. Mineral deposition.
Fukawa et al. (2023) [33]	Human oral squamous cell carcinoma (OSCC) lines co-cultured with osteoclast precursor cells (OPCs) from 5-week-old ddY mouse bone marrow. OPCs generated with M-CSF → RANKL (100 ng/mL). Human OSCC lines: NEM, NEM-F, NEM-K, 3A, Ca, HSC-2/3/6, NA, NU, OMI, SH, Toh. Models: (a) OSCC supernatant exposure (40% supernatant). (b) conventional RANKL (100 ng/mL) osteoclastogenesis.	CBD at 5 µM (other treatment not reported).	n/a.	OSCC culture supernatant-stimulated osteoclast formation. Pit formation. GR55 agonist abolished the effects of CBD.	RANKL-induced osteoclastogenesis (TRAP+ cell counts and pit formation). Cell viability of OPCs.
Kang et al. (2020) [22]	U2OS and MG-63 human osteoblast-like cell lines.	CBD at 0.125, 0.25, and 0.5 µM. Negative control: vehicle only. Positive control: n/a.	Angiopoietin-1, tight-junction protein, ALP protein expression. Cell migration. Mineralisation Expression of osteogenic markers: DLX5 (mRNA), BSP (mRNA), OCN (mRNA), COL1 (mRNA), RUNX2 (protein), OSX (protein). p38 MAPK activation. Protein–protein interaction: RUNX2–OSX–pp38.	n/a.	Angiopoietin-2. Cell morphology normal.
Li et al. (2022) [21]	BMSCs from 5-week-old male C57BL/6 mice; passages 3–5. Inflammatory model: LPS 10 µg/mL.	CBD 0.1–10 µM, with maximal effects at 2.5 µM. Co-treatments included AM630 (10 µM-CB2 antagonist) and SB203580 (20 µM-p38antagonist). Negative control: LPS and vehicle. Positive control: n/a.	ALP production. Proteins and mRNA expression of RUNX2, ALP, and OCN. Mineralisation. CB2 protein expression. AM630 and SB203580 reverse the effects.	TNF-α and IL-6 mRNA expression.	BMSC viability (CBD alone). CB1 protein expression.
Nielsen et al. (2024) [34]	Human osteoclast precursors = CD14^+^ monocytes (>50-year-old male donors). Human osteoblast-lineage cells = trabecular bone outgrowths from OA patients.	CBD 0.3–30 µM during 7–10-day differentiation assays, 3-day resorption assays, and 72 h cocultures. Negative control: vehicle. Positive control: none.	ES/BS, pit surface, trench surface.	Osteoclast fusion. Multinucleated osteoclasts. Bone resorption. Osteoblasts. ALP protein expression Proliferative expansion. (long-term 3 µM).	Percentage trench surface per eroded surface.
Schmuhl et al. (2013) [35]	Human MSCs from liposuction adipose tissue; CD34^+^ selected. Short-term assays in serum-free DMEM; long-term ≤35 days in DMEM + 1% FCS + osteogenic supplements.	CBD 0.01–3 µM. Negative control: vehicle. Positive control: n/a.	MSC migration. MAPK, Akt, FAK, ALP protein expression. Mineralisation. Upregulate osteogenic genes.	IL-1β secretion. NLRP3, ASC, and pro-Caspase-1 protein expression. TGF-β1/Smad2/3 protein expression. Fibrosis markers: HYP, α-SMA, and OPN protein expression. Fibrosis score.	Cell viability.
Tsuchiya et al. (2019) [36]	OPCs from 5-week-old female ddy mouse BMMs (M-CSF + short RANKL). Human OSCC lines: 3A, NEM, HO-1-N1. Model compares osteoclastogenic vs. non-osteoclastogenic OSCC lines.	CBD 1–5 µM, with inhibitory effects observed at ≥4 µM over four days of coculture. Negative control: vehicle. Positive control: denosumab.	n/a.	Osteoclast formation (TRAP+ multinucleated cells). Tumour-induced osteoclastogenesis by 3A OSCC cells.	RANKL-induced osteoclastogenesis. NFATc1 mRNA expression.
Yu et al. (2023) [37]	Human DPSCs from 20 healthy donors (12–20 years). Basal and inflammatory conditions (TNF-α 20 or 50 ng/mL). Osteogenic/odontogenic induction × 28 days.	CBD at 0.1–12.5 µM, with 2.5 µM optimal across assays lasting 4–28 days. Negative control: TNF-α without CBD. Positive control: n/a.	Cell proliferation. ALP protein expression. Mineralisation. Osteogenic/Odontogenic gene expression: RUNX2, COL-I, OPN, CB1/CB2.	Cell viability. TNF-α-induced IL-1β, IL-6 and TNF-α mRNA expression.	RUNX2.
* Chen et al. (2023) [23]	Primary human PDLCs from healthy orthodontic premolars (16–20 y); passages 3–5. Stimulated with 1 µg/mL LPS.	CBD 1–8 µM. Negative control: no CBD. Positive control: n/a.	Cell viability.	Gene expression of TNF-α (2–8 µM), IL-1β (8 µM), TLR4 (2–8 µM). Protein expression of TNF-α (1–8 µM), IL-1β (2–8 µM), TLR4 (1–8 µM) p-NF-κB (1–8 µM), p-ERK (8 µM).	Cell viability (not stimulated by LPS).
* Ihejirika-Lomedico et al. (2023) [16]	Human skeletal stem/progenitor cells (SSPCs) from discarded femoral heads (hip replacement).	CBD 1–10 µg/mL.	Viability and Proliferation. OCN mRNA expression.	n/a.	RUNX2 and Osterix mRNA expression.
* Kamali et al. (2019) [20]	Bone marrow-derived MSCs seeded on a scaffold.	CBD encapsulated into PLGA microspheres and incorporated into a gelatin/nano-hydroxyapatite scaffold. Negative control: Scaffolds without CBD. Positive control: n/a.	MSC migration. ALP, Col1, and OCN mRNA expression.	Fibroblast/fibrocyte density. ALP mRNA (late timepoint).	MSC viability.
* Kim et al. (2025) [38]	RAW264.7 murine macrophages + mouse bone-marrow-derived macrophages (BMMs).	CBD (10 µM) for 30 min before LPS stimulation. Negative control: LPS + vehicle. Positive control: n/a.	n/a.	Only for CBD + taurine: protein expression of iNOS, COX-2, TNF-α, IL-1β. TRAP^+^ osteoclasts. Resorption pit area.	Cell viability (≤ 12 µM CBD).
* Kogan et al. (2015) [11]	Primary newborn mouse calvarial osteoblasts.	CBD at 10^−12^–10^−10^ M for 24 h before gene analysis.	Plod1 mRNA.	n/a.	n/a
* Liu et al. (2024) [39]	Human dental pulp stem cells (DPSCs) grown as 2D monolayers and 3D microspheroids (≈70 µm) using PDMS-agarose microwells.	CBD 0–12.5 µM CBD 2.5 µM during osteogenic induction.	ALP production. Mineralisation. Osteogenic gene and protein expression (for microspheroids): ALP, BMP2, RUNX2, OCN. WNT6, β-catenin protein expression.	n/a.	Cell viability unaffected at 0.1–2.5 µM.
* Whyte et al. (2009) [40]	Human osteoclasts from peripheral blood monocytes; mouse osteoclasts from WT and GPR55^−^/^−^ BMMs.	CBD 0.5 and 1 µM.	Osteoclast number.	Osteoclast polarisation (F-actin rings). Resorption pit area (human osteoclasts). Activated Rho and p-ERK protein expression.	Osteoblast differentiation and mineralisation.

Abbreviations: ALP, alkaline phosphatase; BMM, bone marrow-derived macrophage; BMSC, bone marrow stromal cell; BSP, bone sialoprotein; CB1, cannabinoid receptor type 1; CB2, cannabinoid receptor type 2; CBD, cannabidiol; COL1, collagen type I; DPSC, dental pulp stem cell; ERK, extracellular signal-regulated kinase; GPR55, G protein-coupled receptor 55; IL-1β, interleukin 1 beta; IL-6, interleukin 6; iNOS, inducible nitric oxide synthase; LPS, lipopolysaccharide; MAPK, mitogen-activated protein kinase; M-CSF, macrophage colony-stimulating factor; MSC, mesenchymal stem cell; NFATc1, nuclear factor of activated T-cells, cytoplasmic 1; NFκB, nuclear factor kappa-light-chain-enhancer of activated B cells; OCN, osteocalcin; OPC, osteoclast precursor cell; OPG, osteoprotegerin; OSCC, oral squamous cell carcinoma; OSX, Osterix; PDLC, periodontal ligament cell; RANKL, receptor activator of nuclear factor kappa-B ligand; RUNX2, runt-related transcription factor 2; THC, tetrahydrocannabinol; TLR4, Toll-like receptor 4; TNF-α, tumour necrosis factor alpha; TRAP, tartrate-resistant acid phosphatase; TRPV1, transient receptor potential vanilloid 1. Notes: Studies marked with * have animal components.

**Table 2:** Summary of animal studies on the effects of CBD on skeletal health.

Authors (Year)	Subject and Model Characteristics	Treatment Characteristics	Findings		
			Increased vs. Negative Control	Decreased vs. Negative Control	Unchanged vs. Negative Control
Bradley et al. (2022) [41]	Clinically healthy dogs. Breeds/age/weight: 17 Labrador Retrievers (1.4–9.4 y; 19–36 kg), 8 Beagles (1.2–6.6 y; 11–18 kg), 15 Norfolk Terriers (1.4–4.4 y; 4–8.5 kg).	Treatment (*n* = 20): CBD distillate soft gel capsules at ~4 mg/kg/day (3.38–4.44 mg/kg/day) for 26 weeks. Negative control (*n* = 20): placebo capsules. Positive control: n/a.	ALP and BALP protein expression.	Total protein level. Calcium level.	Liver profile. Serum C-terminal telopeptide. Haematological profile. Urine profile. Quality-of-life measures.
de Oliveira et al. (2024) [17]	OVX Female Sprague–Dawley rats, 8 weeks, ~200 g.	Treatment (*n* = 12): CBD at 5 mg/kg (i.p.), five days per week for three weeks (15 total doses), given 9 weeks after OVX. Negative control (*n* = 12): vehicle. Positive control: n/a.	BMD, BV/TV, Tb.N, Strength, B.Ar/Tt.Ar, Osteoblast count.	Osteoclast count.	Femoral neck BMD. RANKL and OPG mRNA expression. Cortical thickness.
Fogel et al. (2024) [18]	Female Sprague–Dawley rats, 13 weeks. Model: L4–L5 posterolateral inter-transverse lumbar spinal fusion; transverse processes decorticated; graft placed bilaterally.	Treatment (*n* = 18/treatment): CBD, THC, or CBD + THC (NIDA) at 5 mg/kg once weekly for eight weeks. Negative control (*n* = 18): vehicle. Positive control: n/a.	Histology score ALPL, BMP4, and SOST protein expression. Fusion rate.	RANKL/OPG ratio RANK, RANKL protein expression.	Micro-CT-BV/TV, BMD and TMD. mRNA expression (2 weeks): RUNX2 and β-catenin (CTNNB1). Col1A1 and MMP13. Gene expression (8 weeks): ALPL, BMP4 [mRNA] and SOST. RUNX2 & β-catenin (CTNNB1).
Khajuria et al. (2023) [12]	Male C57BL/6J mice, 14 weeks (~30 g). Model: open mid-diaphyseal tibial fracture with intramedullary nail fixation. In vitro: periosteal progenitors of the mice (PDGFRα^+^).	Treatment (*n* = 6): CBD 5 mg/kg/day (i.p.) beginning 24 h after fracture. Negative control (*n* = 6): vehicle. Positive control (*n* = 6/group): indomethacin (2.5 mg/kg) and celecoxib (3 mg/kg) for hypersensitivity test.	Pain thresholds. Gait parameters. Collagen I, OCN, and SP7 staining area. BV/TV, BMD, Tb.Th, Tb.N. No. of osteoblasts. Biomechanics. PDGFRα^+^ progenitors.	No. of TUNEL+ cells.	Inflammatory phase markers (IHH, Col X, MMP13). Bone stiffness.
Li et al. (2017) [19]	Male Wistar rats (~3 months). Thoracic (T3–T4) complete spinal cord transection—severe sublesional bone loss.	Treatment (*n* = 9): CBD 0.5 or 5 mg/kg/day for 14 days, beginning 12 h after spinal cord transection. Negative control (*n* = 9): vehicle. Positive control: n/a.	Only at 5 mg/kg: OCN, ALP, and OPG protein expression. BMD, BV/TV, Tb.Th, Tb.N. Ultimate compressive load, stiffness, and energy to max force of femoral diaphysis. wnt3a, Lrp5, and ctnnb1 mRNA in femurs.	Only 5 mg/kg group: CTX level. Tb.Sp. RANKL. TRAP.	Sost, Wnt1 mRNA. Displacement at the ultimate load.
Napimoga et al. (2009) [24]	Male Wistar rats. Ligature-induced periodontitis (mandibular 1st molars, 30 days).	Treatment (*n* = 10): CBD 5 mg/kg/day (i.p.) for 30 days beginning the day after ligature placement. Negative control (*n* = 10): vehicle. Positive control: n/a.	n/a.	Alveolar bone loss. RANKL, RANK staining. Neutrophil infiltration (MPO expression). TNF-α, IL-1β production.	OPG protein expression.
Sui et al. (2022) [42]	Female C57BL/6J mice; OVX at 12 weeks.	Treatment (*n* = 9): CBD at 25 mg/kg/day for 18 weeks, started 2 weeks after OVX. Negative control (*n* = 8–9): vehicle. Positive control: n/a.	Whole-body BMD, BMC, BV/TV, Tb.Th, vBMD, and CB2/TGR5 genes. O_2_ consumption. EE. *Lactobacillus* in the gut.	Femoral RANKL (Tnfrsf11) mRNA. Femoral IL-6 mRNA.	Acp5 (TRAP), OPG (Tnfrsf11b) and RANK (Tnfrsf11a) mRNA. Tb.N and Tb.Sp. Ct.Ar/Tt.Ar, Ct.Th, and tissue mineral density.
Trivedi et al. (2022) [43]	Male Sprague–Dawley rats. Vitamin D_3_ deficiency induced by VDD diet × 3 weeks (↓25-OH D_3_ by ~50–60%).	Treatment (*n* = 6/group): CBD 15, 30 or 60 mg/kg for 56 days. Negative control (*n* = 6): vehicle. Positive control (*n* = 6): calcitriol (0.5 µg/kg).	CB2 mRNA, VDR mRNA 25(OH)D, and 1,25(OH) in the kidney, liver, and serum (at 60mg/kg) T4. Calcitonin	TSH, PTH	n/a.
* Chen et al. (2023) [23]	Male Sprague–Dawley rats, 8 weeks old. Periodontitis induced via bilateral maxillary first-molar nylon ligature.	Treatment (*n* = 10): CBD (5% w/w) was incorporated into a beeswax/porcine fat paste, applied topically at ~5 mg/kg/day to ligature sites for four weeks. Negative control (*n* = 10): ligature without CBD. Positive control: n/a.	BV/TV. Collagen organisation.	Alveolar bone loss. TNF-α, IL-1β, and TLR4 protein expression. Tissue destruction	n/a.
* Ihejirika-Lomedico et al. (2023) [16]	C57BL/6 mice: Phase 1—12-week-old males; Phase 2—8-week-old females. Bone-loss induction: fluoxetine (10 mg/kg/day) or ovariectomy (OVX). Fracture model: standardised mid-shaft femoral fracture with screw fixation.	Treatment (*n* = 5–7): CBD 5 mg/kg/day for three weeks using osmotic pumps implanted pre- or post-fracture. Negative control (*n* = 5–7): Vehicle pumps + OVX or Fluoxetine. Positive control: n/a.	OVX model, fractured bone: bone formation staining (post-fracture treatment) OVX model, unfractured bone, pre-treatment: Tb.N, BMD Fluoxetine model: BMD after 4 weeks of treatment.	OVX model, fractured bone: Tb. Sp. (post-fracture treatment). Cartilage persistence.	OVX model, fractured bone, pre- and post-fracture treatment: BV/TV, Tb.Th, Tb.N, Tb.Sp (pre-fracture treatment). Bone formation staining (pre-fracture treatment), cartilage area. OVX model, unfractured bone, post-treatment: Tb.N, Tb, Th, Tb.Sp, BMD. Pre-treatment: Tb.Th, Tb.Sp. Fluoxetine model: Stiffness, ultimate stress, ultimate load, elastic modulus. Collagen staining.
* Kamali et al. (2019) [20]	In vivo: Adult male Wistar rats. Model: Bilateral 5 mm critical-sized radial defect	Treatment (*n* = 10): CBD-PLGA-G/nHAp scaffold was implanted into bone defects. Negative control (*n* = 10): Empty defect + scaffold. Positive control (*n* = 10): autografts.	BV/TV. Union (X-ray). Osseous and cartilaginous tissue density. Collagen I, OCN and OPN staining. MSC recruitment. Ultimate load, stress, and stiffness.	n/a.	Scaffold porosity and mechanical properties.
* Kim et al. (2025) [38]	6-week-old male Sprague–Dawley rats with ligature-induced periodontitis (*P. gingivalis*–soaked ligatures, 7 days).	Treatment (*n* = 9): CBD (2 or 20 mg/kg) + taurine (100 mg/kg) p.o. for 14 days. Negative control (*n* = 9): vehicle only. Positive control: n/a.	n/a.	Only for 20 mg/kg CBD + taurine: Serum TNF-α and IL-1β. Distance from cementoenamel junction to alveolar bone crest. Periodontal pocket depth.	n/a.
* Kogan et al. (2015) [11]	Male Sprague–Dawley rats with stabilised unilateral femoral fracture (1.1 mm pin).	Treatment (*n* = 5–13): CBD 5 mg/kg/day (i.p.) after fracture for up to eight weeks. Negative control (*n* = 5–13): vehicle. Positive control: n/a.	Maximal load, work-to-failure. Callus formation. Collagen cross-linking ratio.	Total callus volume at week 4.	Callus material density/mineralisation. Stiffness.
* Liu et al. (2024) [39]	Male nude mice (6–8 weeks, 19–26 g) with 3 mm calvarial defects implanted with GelMA constructs ± CBD-pretreated DPSCs.	Treatment (*n* = 8): GelMA constructs containing CBD-pretreated DPSCs were implanted for eight weeks. Negative control (*n* = 8): GelMA scaffold without cells. Positive control: n/a.	(microspheroids > DPSCs) BV/TV, BS/TV, BMD, Tb.N. Osteoid/bone formation.	n/a.	Tb.Sp, Tb.Th.
* Whyte et al. (2009) [40]	Male C57BL/6 mice (12 weeks) ± CBD.	Treatment (*n* = 5): CBD 10 mg/kg three times/week for eight weeks. Negative control (*n* = 5): vehicle. Positive control: n/a.	BV/TV, Tb.N.	Serum CTX level.	n/a.

Abbreviations: ALP, alkaline phosphatase; BALP, bone-specific alkaline phosphatase; BMD, bone mineral density; BV/TV, bone volume per tissue volume; CB2, cannabinoid receptor type 2; CBD, cannabidiol; COL1, collagen type I; CTX, C-terminal telopeptide; DXA, dual-energy X-ray absorptiometry; EE, energy expenditure; IL-1β, interleukin 1 beta; IL-6, interleukin 6; OCN, osteocalcin; OPG, osteoprotegerin; OVX, ovariectomized; P1NP, procollagen type I N-terminal propeptide; RANKL, receptor activator of nuclear factor kappa-B ligand; RUNX2, runt-related transcription factor 2; Tb.N, trabecular number; Tb.Sp, trabecular separation; Tb.Th, trabecular thickness; THC, tetrahydrocannabinol; TNF-α, tumour necrosis factor alpha; TRAP, tartrate-resistant acid phosphatase. Notes: Studies marked with * have cell culture components.

## Data Availability

Not applicable.

## Supplementary Materials

The following supporting information can be downloaded at: https://www.mdpi.com/article/10.3390/biomedicines14010208/s1, Table S1: PRISMA-ScR checklist.
